# A simple climate change model for the concerned public

**DOI:** 10.14324/111.444/ucloe.3219

**Published:** 2026-06-25

**Authors:** Philip J. Wilson

**Affiliations:** 1 Independent researcher, Kent, UK

**Keywords:** Intergovernmental Panel on Climate Change, public understanding of science, concerned public, science education, global warming, simplicity

## Abstract

The Intergovernmental Panel on Climate Change addresses policymakers with elaborate models and projections of global warming that are difficult for most people to understand. The simplest model is the trend line in the recent global mean annual near-surface warming data, and the simplest projection is to extrapolate the line into the future. Over the 50 years from 1974 to 2023 the trend has been close to linear with a mean rate of increase of 0.20 °C per decade. The 20-year time series (2003–2022) gives the very similar rate of 0.21 °C of warming per decade, but annual updates (2004–2023 and 2005–2024) give rates of 0.24 °C and 0.27 °C per decade, respectively. Amid the high variation from year to year, the trend line is a practical index of the mean near-surface warming at any one time. Projected linearly into the future the trend line for the 2005–2024 time series predicts +1.5 °C and +2 °C in 2029 and 2047, not dissimilar to more complex projections. Such simple exercises are open to almost anyone to understand or to perform for themselves and could help to lessen the large gap between the public perception of climate change and scientific reality.

## Introduction

The desired policy to reduce the consumption of fossil fuels is simple in principle, but in practice it gives rise to much public discourse that is, for various reasons, confusing or misleading. Climate science is also broadly straightforward (if complex in detail), so that an understanding of sophisticated climate models is not needed to have an informed view of climate change [1]. The basic requirement is the ability to interpret graphs that present real-world data [2]. The simplest graph shows the recent trend in the global mean annual near-surface warming, and the simplest projection is the extrapolation of the trend line into the future. Such exercises are open to almost anyone to understand or to perform for themselves, and could help to lessen the large gap between the prevailing perception of climate change and scientific reality [3,4].

Much climate information effectively dates from 2020 (the last Intergovernmental Panel on Climate Change [IPCC] assessment report) [5]. Forster et al. [6] gives an annual update that follows IPCC methods and is therefore to be trusted by all associated parties, but this purpose does not lend itself to easy comprehension by the concerned public. Plainer communication is also desired [7,8].

This paper presents the recent trend in global mean annual near-surface warming in four time series, considers extrapolation of the trend lines, and illustrates the utility of such exercises in informing young people and the concerned public. First, the function of the IPCC as an institution, the definition of the global mean near-surface temperature and the uncertainties of extrapolation are briefly reviewed.

### Intergovernmental Panel on Climate Change

The IPCC was established in 1988 by the United Nations Environment Programme and the World Meteorological Organization to undertake authoritative studies of the climate and climate change, and to be the scientific advisory body to the United Nations. In serving this purpose it has so many collaborators that its findings have the character of a wide consensus, but the IPCC is also a single institutional entity, and as such its output is open to question on three grounds: the size of its assessments, the elaboration of its projections and the presentation of its results.

First, the IPCC’s assessments are so large as to be inevitably conservative [9,10]. Public disagreement that might be used as an excuse for inaction is avoided to the extent possible, so high-end estimates tend to be excluded, and scientists who are outspoken are excluded for similar reasons. Scientists’ reputations are less likely to be harmed by underestimates, an anxiety increased in the field of climate change by the threat of attack by hostile commentators, and if the private views of some collaborating scientists differ from their official views, as they do [11], to air such differences would also risk reputational damage [12]. In addition, the IPCC is subject to lobbying by national governments, some of whom are lukewarm or even antagonistic to the idea of climate change action [13].

Second, for most people, the IPCC’s assessments are too elaborate to be easily understood. Even the *Summaries for Policymakers* are so dense that it is doubtful that policymakers, if unschooled in science, really do read and understand them. They create an air of monolithic authority that sometimes approximates to mystique. Global climate models are so complex that leading modellers are gatekeepers to knowledge about the climate [8], and the associated esoteric language raises the bar for popular participation [14].

Third, the presentation of some of the IPCC’s findings is questionable. Its integrated assessment models, which combine science with economics, omit many big risks [15] and are wishful [16], and very long-term projections to 2100 and even 2200 have a comforting remoteness but do not, as they seem to imply, render the distant future knowable or tractable. Some feedback-related warming, such as that due to the increasing emissions of methane from (warming) wetland, is also excluded from the anthropogenic account [6].

### Global mean near-surface temperature

Global mean annual near-surface temperature is estimated precisely but integrates many diverse climate phenomena and so varies appreciably from year to year. The Arctic is warming up to four times faster than the rest of the world [17]. The near-surface of the land is warming faster than the surface waters of the ocean (exceeding 2 °C and 1 °C of warming, respectively, for the first time in 2023 [18]). Sea surface temperatures contribute to the global mean data rather than the air temperatures near the ocean surface because they are relatively easily measured from buoys and satellites, but the two are warming at slightly different rates [19]. And the temperature of the surface waters is very dependent on the degree to which they mix with deeper (generally colder) waters. Surface warming reduces this mixing [20,21], reducing the amount of heat absorbed by the deep ocean and making marine heatwaves of the surface waters more likely [22]. Numerous climate-related phenomena are unpredictable, cyclical, interrelated or subject to feedback processes, adding to the variation.

International accords and public discourse are dominated by the global mean near-surface temperature thresholds of +1.5 °C and +2 °C above the pre-industrial mean. While no better policy objectives have been proposed, they are arbitrary (for instance, depending on the definition of the pre-industrial mean), and have a static quality that diverts attention from the dynamism of the climate including rates of change.

The IPCC’s method for determining the global mean near-surface temperature is the 20-year running mean [23], the authoritative method for years of interest that have retreated at least 10 years into the past. In a method involving lesser delay, three consecutive years warmer than +1.5 °C would give better than 90% confidence that this threshold had been reached [24], while instantaneous (i.e., non-retroactive) estimates include the mean of the last 10 years combined with model data for the next 10 [23] and inference from a trend line fitted to a time series of annual means, as in this paper. These latter methods are not future-proof nor consistent with existing IPCC practice [23], but a non-retroactive method is clearly needed [25].

### Extrapolation

To extrapolate is to estimate beyond a known range. It is not to be trusted but may have a speculative purpose. The inertia in Earth processes and human society suggests that short-term climate projections are not unreasonable, but longer-term projections, however intricate, are increasingly and inevitably prone to error.

An extrapolation may be linear, upward or downward. Among the upward influences on the rate of global near-surface warming, the increase of carbon dioxide (CO_2_) in the atmosphere has been accelerating on a timescale of decades [26]. The Earth’s ocean heat content [27] and energy imbalance [28] have also been accelerating since the 1960s, and the rate of sea level rise doubled over 20 years from 2.1 mm per year (1993–2002) to 4.8 mm per year (2014–2023) [29].

The global mean near-surface temperature itself also appears to be accelerating on a decadal timescale. It increased by 0.14 °C and 0.21 °C in the decades 1991–2000 and 2001–2010, respectively [30], while the estimated decadal rate 2010–2023 is 0.30 °C [31]. Recent climate models predict 0.29 °C per decade (2015–2050) [32] and 0.32 °C per decade (2011–2050) [33]. Positive climate feedback, such as the loss of albedo as the Arctic ice retreats [34], are expected to contribute to the acceleration under business-as-usual.

Alternatively, warming could slow down as emissions decrease, perhaps owing to policy change such as a carbon tax with border tariff and dividend [35], hoped-for innovation or any measure that constrains growth. However, for 50 years, greenhouse gases in the atmosphere have increased apparently unperturbed by any environmental accord or policy, and this insensitivity suggests that a substantial reduction will only come about through force of circumstance.

## Methods

HADCrut5.2 data, one of several independent datasets of global near-surface temperature [19], were downloaded in January 2025 [36]. The mean global near-surface temperature for 1850–1899 was calculated (the pre-industrial mean), and the difference between that value and each of the last 50 years of global mean annual near-surface data was determined as the temperature anomaly (i.e., the amount of warming). Simple linear regression lines were fitted using the statistical software INSTAT, University of Reading.

The anomaly data were plotted to show the trends in global warming over the last 50 years (1974–2023) and in three 20-year time series (2003–2022), (2004–2023) and (2005–2024).

## Results

**Figure 1:**
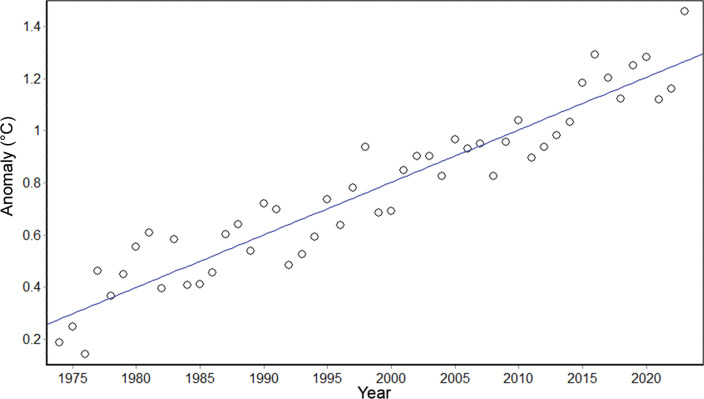
Global warming as the temperature anomaly (global mean annual near-surface temperature minus the pre-industrial mean) over the last 50 years (1974–2023). The Earth has warmed approximately linearly, as the fitted line emphasises.

**Figure 2:**
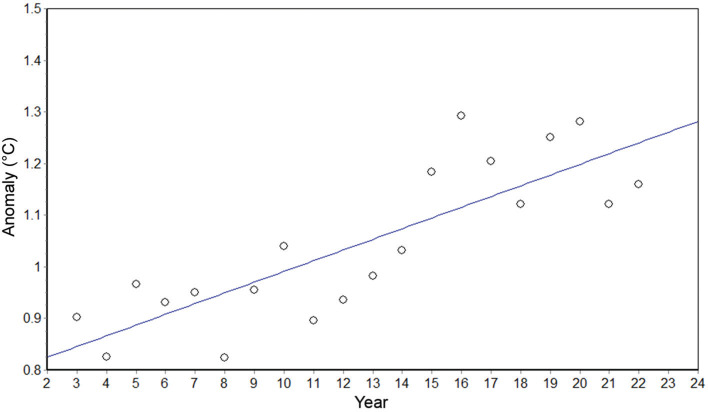
Global warming as the temperature anomaly (global mean annual near-surface temperature minus the pre-industrial mean) over the 20 years 2003–2022. The trend line gives a similar rate of warming to that in Fig. 1, about 0.21 °C of warming per decade compared to 0.20 °C.

**Figure 3:**
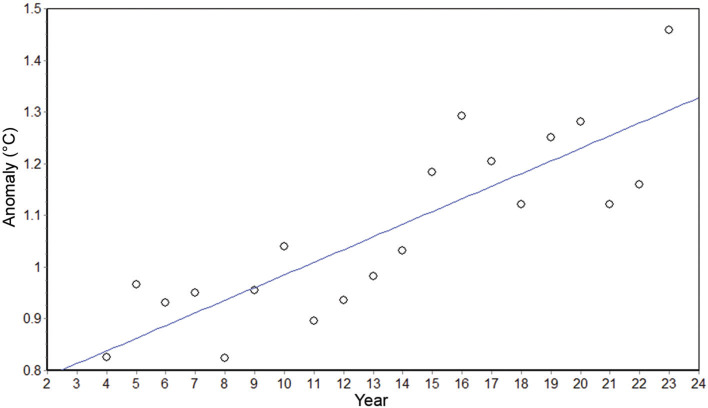
Global warming as the temperature anomaly (global mean annual near-surface temperature minus the pre-industrial mean) over the 20 years 2004–2023. The trend line, now including the high outlier for 2023, gives a rate of warming of 0.24 °C per decade.

**Figure 4:**
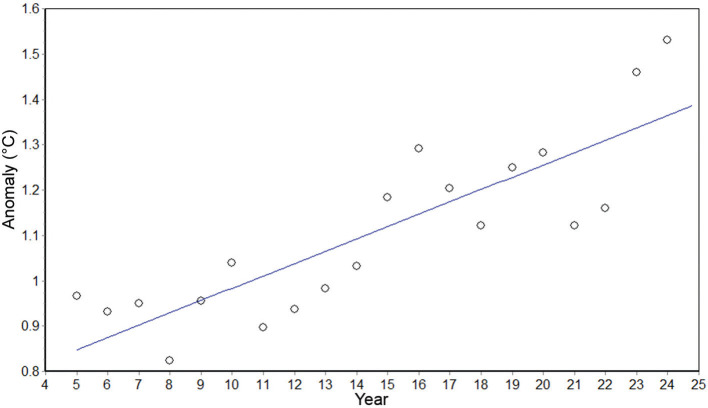
Global warming as the temperature anomaly (global mean annual near-surface temperature minus the pre-industrial mean) over the 20 years 2005–2024. The trend line, now including the two high outliers of 2023 and 2024, gives a rate of warming of 0.27 °C per decade.

The trend lines shown in the figures are the linear:

1. Anomaly = (0.0202 × Year) − 39.59       r^2^ = 0.90 (Fig. 1; 50 years 1974–2023)
2. Anomaly = (0.0208 × Year) + 0.7835   r^2^ = 0.68 (Fig. 2; 20 years 2003–2022)
3. Anomaly = (0.0245 × Year) + 0.7401   r^2^ = 0.72 (Fig. 3; 20 years 2004–2023)
4. Anomaly = (0.0274 × Year) + 0.7091   r^2^ = 0.72 (Fig. 4; 20 years 2005–2024)

Figure 1 shows that the Earth warmed approximately linearly over the 50 years to 2023 at the rate of 0.20 °C per decade. In this time series the datum for 2023 is seen to be a high outlier but comparable to the earlier high outliers of 2016 and 1998 (all El Niño years), although in more sensitive models the 2023 datum was 0.2 °C higher than predicted [37].

The 20-year time series 2003–2022 (Fig. 2) has a very similar rate of warming (0.21 °C per decade) to that of Fig. 1, but updating the 20-year series by one year (2004–2023) increases the rate to 0.24 °C per decade (Fig. 3), and a further update of one year (2005–2024) gives 0.27 °C per decade (Fig. 4).

### Linear projections

In the linear extrapolation of the 50-year data, the world reaches +1.5 °C and +2 °C in 2034 and 2059. The trend lines of the progressively updated 20-year time series increase in steepness, the linear extrapolation of the most recent (2005–2024) giving +1.5 °C and +2 °C in 2029 and 2047. This is not dissimilar to the 2031 and 2043 predicted in a high-emissions scenario [38], or the 2032 and 2052 predicted for an ensemble of models [39].

The linear extrapolation of the 2005–2024 trend line predicts an annual mean of +1.39°C for 2025, not dissimilar to the Met Office’s central estimate of +1.41 °C [40]. Referring to Fig. 4 and envisaging the 20-year data 2006–2025, this implies a further increase in the rate of warming (to 0.29 °C per decade; data not shown).

Annual updates (as in Figs 3 and 4) are very easy to perform but are to be treated with caution. They are helpful in showing rates of change, supplementing the attention generally given to annual means and the static (and somewhat thought-terminating) thresholds of +1.5 °C and +2 °C. If near-surface warming were to remain at +0.3 °C per decade, +3 °C would be reached in 2078.

### Non-linear projections

According to a mainstream view, despite the influence of El Niño, the outlying warmth of 2023 is still largely unexplained [18]. It has been attributed to a loss of low-level cloud cover and consequent lowered albedo owing to a reduction in aerosol pollution, some other climate feedback or natural variability [38]. Hansen et al. [2] also favoured a reduction in sulphur aerosols and associated cloudiness, especially over the ocean owing to cleaner shipping fuels. This effect may have been at least partly negated by an increase in aerosols due to wildfire [6], whose emissions in the 2023–2024 fire season, at least of CO_2_, were 16% higher than the 20-year average [41].

Amid the high variation from year to year, it is still early to infer a recent acceleration from Figs 2 to 4, but in due course, with further annual updates of the 20-year time series, an acceleration could be reasonably inferred without having to rely on a non-linear model.

## Discussion

Caution is required in choosing the variables to correlate in simple linear regression, and in inferring cause and effect. The global mean near-surface temperature is a very imperfect index of the Earth’s energy imbalance but is relatively directly associated with the impacts on humanity and is the variable most often referred to in public discourse. Linear trend lines in time series of the global annual near-surface means are shown to be informative, and simple projections of them approximate to the output of more complex models.

The global mean near-surface temperature (across years) at any one time will attract increasing attention as the +1.5 °C threshold approaches. A trend line is an index of this mean, is easily understood and is based on real-world data. For most purposes it is more practical than the IPCC’s 20-year running mean [23], which is the definitive statistic but is only applicable to data that have retreated at least 10 years into the past. A trend line projected into the future is also a simple way of estimating when a particular global mean will be reached.

In applying the rudimentary methods employed in this paper, a consensus on the length of the time series to be studied would be desirable. More years add to statistical confidence (cf. Figs 1 and 2), but only if the model is good over the whole range. If a straight-line model is preferred while still accepting the possibility of acceleration a relatively short time series would be indicated, when a 20-year series, as in Figs 2–4, would seem to be a reasonable compromise.

The IPCC as an institution is concerned to avoid disputation that would assist those interests vested in climate delay, and to this extent its conservatism and air of authority are advantageous. Another often-mentioned justification for conservatism is the notion that optimism overcomes fatalism and encourages positive behaviour change [42], but this is disdainful of the public and disfavours intellectual openness [43]. Some authors play down otherwise alarming evidence perhaps to be reassuring. In mid-2024, 6% of IPCC authors responded that warming could still be limited to +1.5 °C [44], but such hopefulness must sooner or later give way to expressions of surprise, resulting in exactly the public disagreement that is best avoided. Thus, the warming of 2023 has been described in a popular article as ‘entirely predicted’ [45] or alternatively has ‘come out of the blue’ [46].

In interpreting such differences of view a clear distinction is to be made between global mean near-surface warming and the many other changes to the Earth system that have been occurring more quickly than expected. For instance, even 20 years ago it was noted that the ice sheets had begun disintegrating more quickly than expected (Richard Alley cited in [47]), and in relation to the weather extremes of 2023 Dr Caroline Holmes of the British Antarctic Survey said: ‘We don’t really understand the pace of change … we’ve fallen off a cliff without knowing what’s at the bottom’ [48]. Global mean near-surface warming is a conservative index of the Earth’s heat imbalance.

The terms of reference of the IPCC are to inform climate policy, but over several decades this policy has had no discernible effect on the increase in greenhouse gas emissions. The policy is widely regarded as faulty [44], and in deferring to it IPCC authors have been admonished for forgoing their academic independence [49] or for quiescence amounting to irresponsibility [16,50]. However, as the IPCC addresses policymakers, support for policy is a *sine qua non* of participation. If former IPCC insiders or respected outsiders dissent it has little influence on mainstream public discourse, and under these circumstances it would be helpful if a large and trusted institution other than the IPCC [11,25] introduced a reporting procedure specifically for the concerned public.

Young people and the concerned public have long had a poor understanding of the seriousness of climate change [3], but to exert wholesome influence on science-related policy they must be reasonably well informed [51]. Projections so simple that almost anyone can understand or perform them for themselves foster critical thinking, reduce reliance on argument from authority and increase disquiet for a future shaped by climate change.

## Conclusion

The reports of the IPCC are difficult for most people to understand. Simple linear regressions of global mean annual near-surface warming are shown to be reasonable approximations to more complex models and projections. The trend lines give a practical estimate of the global mean (across years) at any one time and are easily updated, increasing awareness of rates of change. The return to simplicity could help young people and the concerned public to observe and interpret the evidence of global warming for themselves, lessening the gap between the prevailing perception of climate change and scientific reality.

## Data Availability

All data generated or analysed during this study are included in this published article.
